# Detection Techniques for Lead Ions in Water: A Review

**DOI:** 10.3390/molecules28083601

**Published:** 2023-04-20

**Authors:** Dan Wu, Yinglu Hu, Huan Cheng, Xingqian Ye

**Affiliations:** 1 National-Local Joint Engineering Laboratory of Intelligent Food Technology and Equipment, Zhejiang Key Laboratory for Agro-Food Processing, Integrated Research Base of Southern Fruit and Vegetable Preservation Technology, Zhejiang International Scientific and Technological Cooperation Base of Health Food Manufacturing and Quality Control, Fuli Institute of Food Science, College of Biosystems Engineering and Food Science, Zhejiang University, Hangzhou 310058, China; 2 Zhejiang Lohand Environmental Technology Co., Ltd., Hangzhou 310018, China

**Keywords:** lead ion, detection techniques, water, wastewater, spectroscopy

## Abstract

Lead pollution has increasingly become the focus of environmental pollution, which is a great harm to the ecological environment and human health. Strict control of the emission of lead pollutants and accurate monitoring of lead are very important. The lead ion detection technologies are introduced here, including spectrophotometry, electrochemical method, atomic absorption spectrometry, and other detection methods, and the methods’ applicability, the advantages, and disadvantages are discussed. The detection limits of voltammetry and atomic absorption spectrometry are as low as 0.1 μg/L, and those of atomic absorption spectrometry are as low as 2 μg/L. The detection limit of photometry is higher (0.01 mg/L), but this method can be achieved in most laboratories. The application of different extraction pretreatment technologies in lead ion detection is introduced. The new technologies develop at home and abroad, such as precious metal nanogold technology, paper microfluidic technology, fluorescence molecular probe technology, spectroscopy, and other emerging technologies in recent years, are reviewed, and the principle and application of various technologies are expounded.

## 1. The Hazard of Lead Ion Pollution

Lead is the most abundant heavy metal in the earth’s crust, which has stable chemical properties, excellent ductility, and easy to form alloys with other metals. Lead can absorb X, γ, and other rays, so it is widely used in industry. The universality of lead use also leads to the universality of lead pollution in our environment. It is a toxic heavy metal, and there has been a lot of research on environmental safety. It is a pure toxic, neurotoxic, heavy metal that has no metabolic benefits and is easily absorbed by the human body, especially through the ingestion of contaminated food or water. There has been sufficient evidence to prove that the toxic effect of excessive intake of lead on humans, especially children, is enormous [1]. Even a small amount of lead that enters the environment should be controlled. However, the discharge of three wastes from lead industry, the use of common daily products, such as lead-containing ceramics/ink/cosmetics, automobile exhaust emissions, and the consumption of lead-contaminated food, have all led to the potential safety hazards of unconscious lead intake in our daily life, which threatens people’s health. Lead pollution is widespread in our daily life [2,3,4]. Wang et al. [5] found in a comparative survey on food intake and lead exposure of Chinese residents that people are most exposed to lead in beverages, followed by dried beans and dark vegetables. Even if the percentage of lead intake is small, it means that lead accumulates in the body all the time through the food people eat every day. With the improvement of people’s requirements for the quality of life, the detection and analysis of lead has been paid more attention.

Lead is usually absorbed by human body or animals and plants in the form of lead ions, then it reacts with biological macromolecular affinity sites in biological systems, affecting different organs of organisms with acute or chronic toxic effects. The heavy metal lead cannot be degraded by organisms and is extremely difficult to discharge, so it accumulates in living organisms. Simultaneous exposure to two or more heavy metals can also have a cumulative effect. Lead is a neurotoxic substance, which is mainly absorbed through the respiratory tract, digestive tract, and skin, and it is rapidly distributed to organs and tissues of the whole body after entering the blood. Therefore, lead is a toxic heavy metal that can lead to systemic diseases of the human body, such as central nervous system damage, lung dysfunction, anemia, cardiovascular dysfunction, etc., and even has certain carcinogenicity [6,7]. Most of the lead in the human body is mainly stored in the bone in the form of trilead phosphate, and a small amount is stored in the liver, spleen, brain, and other organs and cells, and maintains a dynamic exchange process with blood at any time. Therefore, blood lead concentration is usually used in medicine to reflect the health hazards of lead in the human body. The blood lead content of the human body is between 0 and 99 μg/L. The median blood lead of male and female in eastern China is 44.00 (29.00–62.16 μg/L) and 37.79 (25.13–54.35 μg/L), respectively [8]. The international level of concern for human lead poisoning is 100 μg/L. When the contamination of lead in soil reaches a certain degree, it will have a large impact on the growth and quality of plants. High concentrations of lead will inhibit the germination of plant seeds and the growth of seedlings, reduce the content of chlorophyll in plant leaves, resulting in the decline of plant photosynthesis, and affect plant growth and fruit yield [9,10,11,12].

In view of the harmfulness and destructiveness of heavy metal lead ions to the environment, it is of great practical value and scientific significance to use effective technology to remove heavy metal lead from the environment. At present, many methods have been used to remove heavy metal ions in sewage, such as precipitation method, adsorption, electrochemical methods, etc. [13,14,15] Among them, the adsorption method is the most commonly used method and is considered to be one of the most promising methods because of its advantages of simple design, low cost, high efficiency, and easy operation [16,17,18]. Since lead is ubiquitous in our living environment, it is essential to detect lead ions in order to effectively avoid or reduce lead intake. How to quickly and accurately detect the content of lead ion in aqueous solution is a topic actively explored by relevant industry personnel, which is of great significance for people to avoid significant economic losses in agricultural planting and maintain personal health.

## 2. The Traditional Analysis Method for Lead Ion

The traditional analysis methods include the atomic absorption method, inductively coupled plasma emission spectrometry, mass spectrometry, stripping voltammetry, etc. Usually, the methods selected according to different samples, different lead concentration, and different detection limits of analysis methods. Take the lead ion detection method in China’s national standard for drinking water as an example, there are seven analysis methods [19]. The atomic absorption method has the advantages of low detection limit, high accuracy, good selectivity, less sample consumption, and a wide range of applications. It is suitable for the analysis of trace components in samples. Compared with non-flame atomic absorption method, direct inhalation flame atomic absorption spectrophotometry has higher precision, faster determination speed, and less interference, and it is suitable for the determination of lead concentration in various wastewater [19,20,21,22,23,24,25]. The extraction/ion exchange flame atomic absorption method is suitable for clean and surface water [20,21,25]. Although graphite furnace atomic absorption method is highly sensitive, it is highly interfered with by a matrix and is suitable for the determination of lead concentration in clean water [22]. The oscorography polarography also has high sensitivity and wide detection range, its detection limit is about 0.02 mg/L [19]. Under normal circumstances, the pollution concentration of lead in water is not high, so the electrochemical method, atomic absorption method, and inductively coupled plasma emission spectrometry/mass spectrometry are common. Atomic fluorescence analysis is fast, and its cost is lower than that of inductively coupled plasma emission spectrometry/mass spectrometry, but nitrogen has a harsh requirement on the acidity of the system. For samples with high lead concentration, dithizone colorimetric method can also be used, its detection limit of lead concentration is 0.01 mg/L [19]. Comparison of traditional detection methods for lead ions is shown in Table 1.

## 3. New Lead Ion Detection Techniques

The development of lead ion detection methods introduced in the national standard has been relatively mature, and each method has its own advantages and disadvantages. Atomic absorption methods and electrochemical methods can satisfy the detection of lead ion in most cases. However, for laboratories that do not have such expensive instruments, the dithizone colorimeter is a test method that is easier to achieve in ordinary laboratories. The colorimetric method is the only colorimetric spectrophotometry introduced in the national standard method and its operation is complicated. The toxic and easily manufactured properties of trichloromethane also do not apply to some sites that require rapid testing. Therefore, in addition to the test method recommended by the national standard, a variety of more environmentally friendly, more simple, more accurate emerging detection technologies are also developing in the direction of diversification.

### 3.1. Application of Different Extraction Techniques

Due to the low content of lead in actual samples and certain matrix interference effect in the analysis process, certain pretreatment and lead enrichment processes are usually required. For example, in the national standard GB/T 5750-2006, the reaction products of dithizone-lead in water samples were extracted by chloroform solution in colorimetric method [19]. This method belongs to liquid-liquid extraction technology, it has good selective extraction and enrichment effect on lead. In the national standard DZ/T 0064.20-2021, the lead ions in the water samples were enriched by chelating resin and then determined by atomic absorption spectrophotometry [21], it belongs to solid phase extraction technology. Traditional liquid–liquid extraction technology usually needs to use a large dose of organic reagents, which has a large pollution to the environment and a limited enrichment ratio. Based on this, researchers explored other green, economical and safe extraction methods for trace lead detection

Decentralized liquid-liquid microextraction (DLLME) is a new extraction method developed in recent years. It is different from the traditional liquid-liquid extraction method in the national standard method, it has the advantages of less extraction agent consumption, fast extraction time, and high enrichment efficiency. DDLME technique can be used in combination with atomic absorption method, spectrophotometry, and other analytical techniques to determine trace lead in samples. Zhang et al. [26] used light solvent octanol as extraction agent, methanol as dispersing agent, and diethyl dithiocarbamate (DDTC) as chelating agent to establish a dispersive liquid–liquid microextraction-graphite furnace atomic absorption spectrometry (LDS-DLLMEGFAAS) method. The method has a concentration of 87 times lead and a detection limit of 0.15 μg/L. It has been successfully applied to the detection of trace Pb in tap water, drinking water, and South Lake water. He et al. [27] used 8-hydroxyquinoline (8-HQ) as the coordination agent, carbon tetrachloride as the extraction agent and acetone as the dispersible agent to determine trace lead in tea with spectrophotometry, and the detection limit reached 0.045 μg/L. Tang et al. [28] used 2-[(5-bromo-2-pyrene-)]azo-5-diethylaminophenol as the chelating agent, carbon tetrachloride as the extractant, ethanol as the dispersant. The enrichment ratio was up to 94 times, and the detection limit of the method was 0.1 mg/L. DLLME technology usually requires the use of both extractant and dispersant. However, Franca et al. [29] for the first time used non-toxic and biodegradable dimethyl carbonate (DMC) as both extractant and dispersive agent for lead determination, and used dithiophosphate as complexing agent, combined with UV spectrophotometry. The enrichment factor of this method was 8.8, and the detection limit was 0.2 mg/L.

Cloud point extraction(CPE) technology is a new environmentally friendly extraction technology that does not use organic solvents. It is an extraction method based on the solubility and turbidity of aqueous solution of surfactant micelles. This method achieves the purpose of separating the target analytes and sample by controlling the change of reaction conditions, such as pH and temperature. Mao et al. [30] established a method of crown ether double turbidity point extraction (DCH18C6-DCPE) to determine trace lead in environmental water samples and food. Lead was selectively extracted by DCH18C6 to form a hydrophobic complex into L64 enrichment phase, then the L64 enrichment phase obtained by turbidity point extraction was complexed with EDTA solution and extracted into the aqueous phase. The enrichment multiple of this process was 18, the extraction time was 10 min, and the method detection limit was 2.8 μg/L.

Solid phase extraction (SPE) technology has the advantages of simple operation, low organic reagent consumption and low price, and is also widely used in the field of environmental and biological sample analysis. Zhao et al. [31] prepared a novel amine-functionalized polyacrylonitrile and a self-made solid phase extraction column to enrich lead ions in water and successfully determined the lead content in environmental water by combined with inductively coupled plasma mass spectrometry technology. Under the optimized conditions, 95% adsorption rate can be achieved in only 10 min with high selectivity and a detection limit of 2.5 μg/L. Liang et al. [32] synthesized lead ion-imprinted polymer microspheres by ion-imprinted polymerization technology. Additionally, it filled the polymer microspheres into solid phase extraction columns to enrich lead ions in the samples. The extraction column has a maximum enrichment ratio of 250 times and can be reused for more than 12 times. Combined with microwave plasma emission spectrometry, the detection limit of this method is 0.26 μg/L under optimal extraction conditions. Khoshhesab et al. [33] synthesized a magnetic nano-adsorbent nickel ferrite with high adsorption performance for lead ions in water. The extractant can be quickly separated under the external magnet, then eluted with hydrochloric acid solution, and determined by spectrophotometry. The extractant can be reused at least three times. Wang et al. [34] synthesized manganese tetroxide nanoparticles for solid phase extraction of lead, and combined with inductively coupled plasma mass spectrometry to determine lead content in vegetables, the detection limit of which was 4 ng/L. The extraction column has good stability in a weakly acidic environment and can be reused 60 times. Solid phase extraction technology is mostly in conjunction with on-line technology. In addition to its good selective adsorption performance for lead ions, the reuse performance of extraction column is also one of the important factors to evaluate the applicability of the method. Therefore, it is necessary to increase the reuse times of extraction columns in the development of new extraction columns.

Ionic liquid, as a new functional material, is also widely used in the field of extraction, which has the advantages of non-volatilization, high stability, and design of results. Fan [35] prepared three ionic liquids with different cationic side chain lengths [CnMIM] (*n* = 4, 6, and 8), used them as extractants, and used dithizone as chelating agent. The extraction rate of lead ions was higher than 93.5% under optimized conditions, and the ionic liquids can be back extracted by controlling the pH value.

### 3.2. Precious Metal Nanotechnology Applications

**Figure 1:**
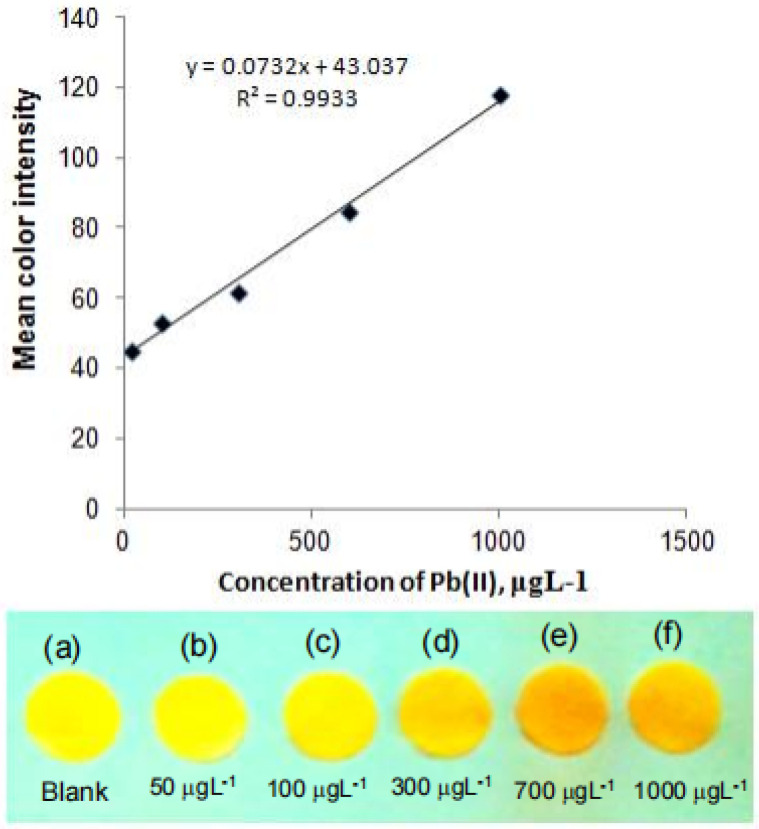
Imaging color on PADs at different lead ion concentrations (**a**–**f**) and the calibration curve between different concentrations of lead from 50 to 1000 μg L^−1^ against the respective mean color intensity [39].

Due to the unique optical properties, silver/gold/copper and other noble metal nanoparticles have been widely used as chemical probes or sensor probes for the analysis of various components in environmental and food samples in recent years [36,37,38]. From this, Shrivas et al. [39] reported a paper-based lead ion analysis device. The device is modified with polyvinyl alcohol (PVA) coated silver nanoparticles (AgNPs). The red shift of the local surface plasmon resonant absorption band occurs when the lead ion touches AgNPs/PVA (as shown in Figure 1). The color intensity of PADs is recorded with a smart phone, then processed with ImageJ software. A new colorimetric method is finally established. The calibration curve has a good linear relationship in the range of 20–1000 μg/L, and the limit of detection (LOD) is 8 μg/L.

**Figure 2:**
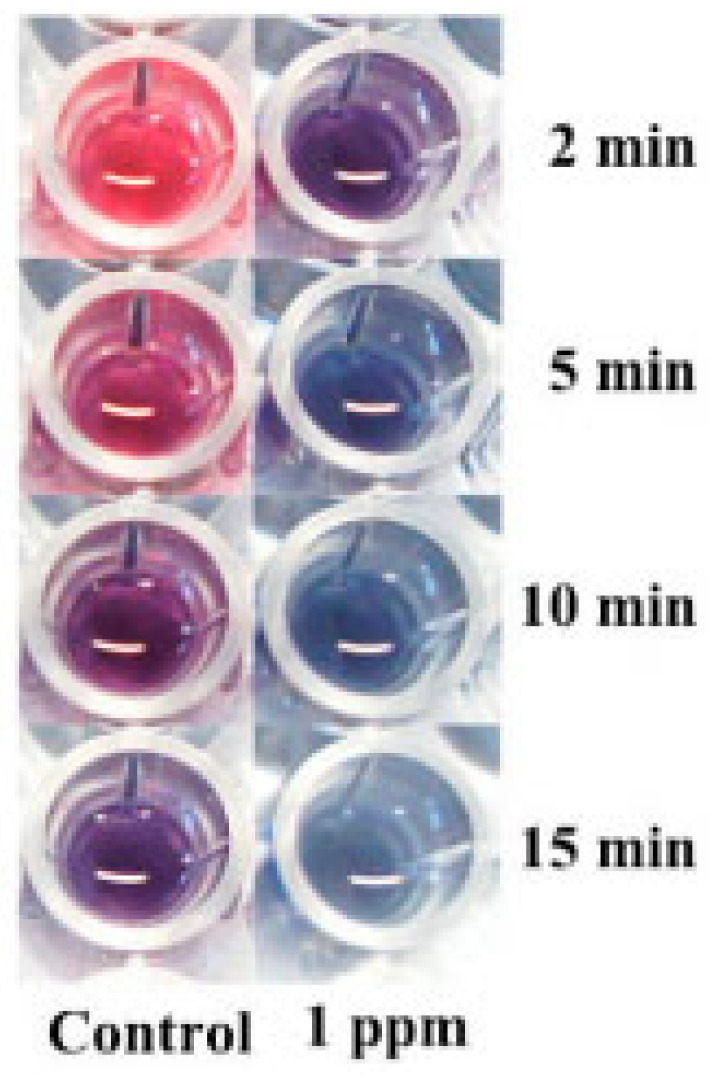
The color of the solution varied with different reaction times [41].

Krian [40] prepared an alumina material coated with 2-Mercaptosuccinic acid (MSA)-capped gold nanoparticles and used the material as a solid extraction agent for lead. The limit of detection was 0.22 μg/L under the optimal conditions. Although the material has a good enrichment and adsorption effect on lead, its specific selective performance for lead is not optimistic, and it is greatly interfered by Zn^2+^, Cl^−^, and SO_4_^2−^. So, the accuracy of the material used for testing lead in actual water samples remains to be studied. Zhong et al. [41] used glutathione (GSH) and gold nanoparticles (AuNP) for the rapid determination of lead. GSH, as a binding agent between lead ion and AuNP, enabled AuNP to selectively bind with lead ion. AuNP rapidly aggregated under the combined action of lead ion and GSH and a rapid color change can be observed. The AuNP solution changed from ruby red to blue within 10 min and the rate of color change was different with different concentrations of lead (as shown in Figure 2). The absorbance ratio between the two colors (A610/A520) has a linear relationship within a certain range of lead concentration, achieving a linear calibration curve of up to 500 ppb and a detection limit of 6 ppb. However, the interaction between the thiol (-SH) groups and the lead cation is usually nonspecific and susceptible to interference by other heavy metals. Due to the well reaction and strong specific selectivity between lead ion and phenolic hydroxyl/carboxyl groups, Berlina [42] et al. synthesized colloidal gold nanoparticles using sodium citrate and sodium tannate as reductants and stabilizers. The corresponding concentration relationship was established by comparing the absorbance changes before and after the interaction between colloidal gold and lead ions at 595 nm. No specific reagent was added to coupling the nanoparticles to the target reactants in this study, and the quantitative limit of the method was 60 ng/mL.

### 3.3. Paper-Based Microfluidic Technology

Although traditional laboratory methods and instruments can achieve accurate quantitative analysis of trace lead in water samples, they often have the disadvantages of complicated operation process, large amount of reagents, expensive analytical instruments, and higher requirements for professional and technical personnel. Paper-based microfluidic technology takes paper as the substrate of microfluidic devices. Various 2D and 3D microfluidic channels can be established through the pore structure and pore size distribution of the paper itself, so that the fluid can be controlled to flow in the pre-designed channel to achieve the effect of detection and analysis. As a low-cost point-of-care diagnostic device, it has the advantages of being easy to carry and a fast analysis speed, which is of great significance for online testing and is widely used in chemistry, biology, medicine, and other fields [43,44,45]. Smartphones also provide an attractive platform for analytical devices for different areas, such as rapid diagnostics and environmental monitoring. Since the smartphone camera is a good color imaging sensor, most of the analysis methods on smartphones are based on colorimetry and macroscopic feature imaging. Additionally, the use of the smartphone camera can be further extended by adding add-on accessories.

**Figure 3:**
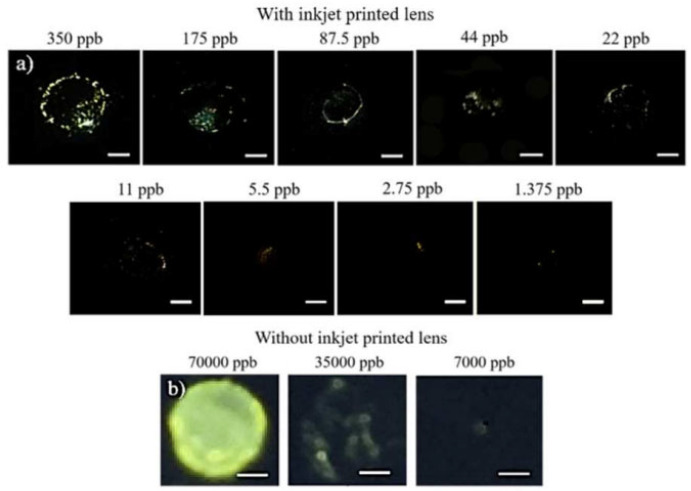
(**a**) PbCrO_4_ sediment imaged by dark-field smartphone microscope with Pb^2+^ concentration ranging from 1.375–350 ppb. The brightness and contrast of the PbCrO_4_ sediment images at Pb^2+^ concentration of 1.375–2.75 ppb was adjusted for display purpose; (**b**) PbCrO_4_ sediment taken by the same smartphone without the objective lens. The yellow color of PbCrO_4_ can only be detected at a concentration above 35,000 ppb. The images are highly blurred compared to the microscopy images [46].

Nguyen et al. [46] established a nano-colorimetric method using smart phones for quantitative detection of soluble lead ions in drinking water based on the principle of quantitative reaction between chromate ions and lead ions to produce bright yellow precipitates. The laboratory has designed a smartphone microscope that can operate in both fluorescence and dark-field imaging modes and enables color detection and intensity quantification at the nanometer level with the smartphone microscope. The sum of the intensities of the yellow pixels showed a good linear relationship with the Pb^2+^ concentration in deionized water (1.37–175 ppb) and in urban tap water (5–175 ppb) (Figure 3a). The same smartphone without the improvement could only detect Pb^2+^ at a concentration of more than 35,000 ppb, and these images were highly blurred compared with microscopic images (Figure 3b).

Satarpai et al. [47] prepared a paper-based lead ion enrichment and concentration material using filter paper as the substrate and zirconium silicate as the adsorption material. It was prepared by transferring a solution of zirconium silicate onto filter paper, then drying it. The modified filter paper was cut into suitable small discs and placed in a simple enrichment device (a micro-centrifuge tube equipped with a peristaltic pump) to enrich lead ions in water. Then, the filter paper, after the lead ion enrichment was dried, was placed in a special detection area device and sodium rodiate was added to react with lead ion to produce a pink substance. The higher the concentration of Pb(II), the stronger the color. The lead ion concentration was determined by taking pictures with a smart phone and processing images with ImageJ image processing software. The detection limit of the method was 10 μg/L, which could realize the detection of 10–100 μg/L lead ion in environmental water.

**Figure 4:**
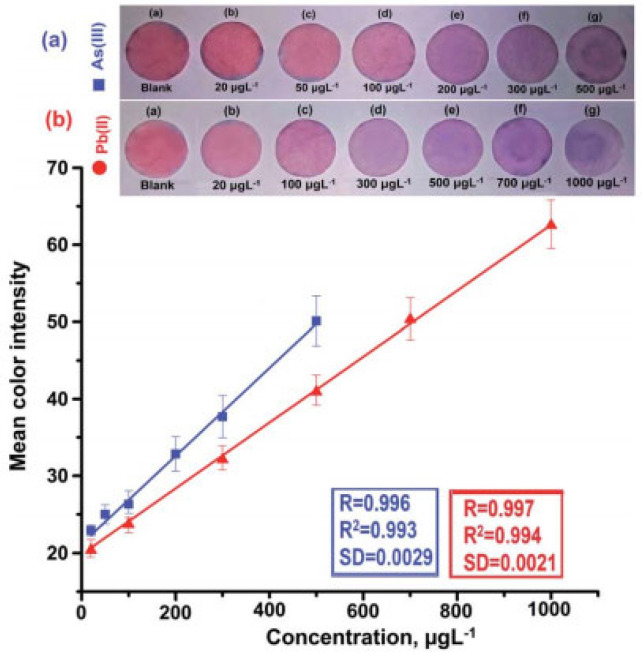
(**a**) Circular filter paper strip fabricated with AuNPs/Glu along with the deposition of different concentrations of As(III) with their calibration curve; (**b**) circular filter strip fabricated with AuNPs/Glu along with the deposition of different concentrations of Pb(II) with their calibration curve [48].

Sahu et al. [48] prepared a paper colorimeter device based on glucose-functionalized gold nanoparticles (AuNPs/Glu) for simultaneous determination of As(III) and Pb(II). The non-covalent interaction between As(III) and Pb(II) and glucose molecules leads to the aggregation of metal nanoparticles, which causes the color change and red shift of the localized surface plasmon resonance (LSPR) absorption band of AuNPs/Glu in the 200–800 nm region. The red shift (Dl) of the LSPR band of As(III) is 525–660 nm and that of Pb(II) is 525–670 nm. A smart phone and ImageJ image processing software were used to process and determine the concentration of those two ions. The color changes after the reaction of different concentrations of ions and the linear relationship are shown in Figure 4.

### 3.4. Spectrometry

The traditional plasma emission spectrometry and atomic absorption spectrometry for the determination of sample lead content require some pre-treatment processes, such as sample digestion and regular calibration of the working curve, which leads to a large amount of time to test a sample. Su et al. [49] used energy dispersive X-ray fluorescence spectrometry to determine lead in polymer materials. The test process did not need to destroy the sample, but only needed to crush the large polymeric sample to a particle diameter of less than 4 mm, which greatly saved the sample pre-treatment time and reduced the detection cost. The detection limit of the method was 4.1 μg/g, and the accuracy was good. He et al. [50] combined a self-designed new type of hydride generator with inductively coupled plasma atomic emission spectrometer and added a self-made pre-stabilizer to the reducing agent to solve the problem of instability of the traditional hydride measurement system and improve the sensitivity of the method. The detection limit of the method can reach 1.0 μg/L, and there is no matrix effect interference. Lehmann et al. [51] studied CO_2_ laser induced spectroscopy for the detection of lead in the classification of industrial recycled glass. Compared with the most advanced X-ray fluorescence technology, the detection limit, detection speed, and detection accuracy of CO_2_ laser-induced spectroscopy are comparable, and the hardware of CO_2_ laser-induced spectroscopy has a lower price and is expected to replace X-ray fluorescence spectroscopy to realize the detection of lead in glass. Tian et al. [52] used Fourier transform infrared spectroscopy based on principal component analysis (PCA) and partial least squares (PLS-DA) to study the biochemical changes in plasma during acute lead poisoning (ALP) in rats. The researchers first collected a large number of plasma samples from rats with and without ALP and found that the corresponding biochemical changes between plasma and lead can be used as potential spectral biomarkers for the diagnosis of lead poisoning. This is the first application of FTIR spectroscopy based on stoichiometry. Arif et al. [53] used a FieldSpec-3 portable handheld ground object spectrometer to measure cadmium (Cd) and lead (Pb) content from 23 roads in the municipality of Chongqing, China. For the Pb content inversion models, the PLS model processed by SG-MSC had the best prediction accuracy. The results of this study are of great value for the portable detection of lead in green space by hyperspectral imaging.

### 3.5. Fluorescent Molecular Probe

Fluorescent molecules can emit a certain wavelength of fluorescence when they are irradiated by ultraviolet or visible light. Its fluorescence properties change with the change of the environment, so as to realize the effective detection of the tested substance. Fluorescence sensor technology has the advantages of good sensitivity, high selectivity, and short response time. In recent years, fluorescence sensors related to heavy metal detection have also been widely concerned by researchers.

**Figure 5:**
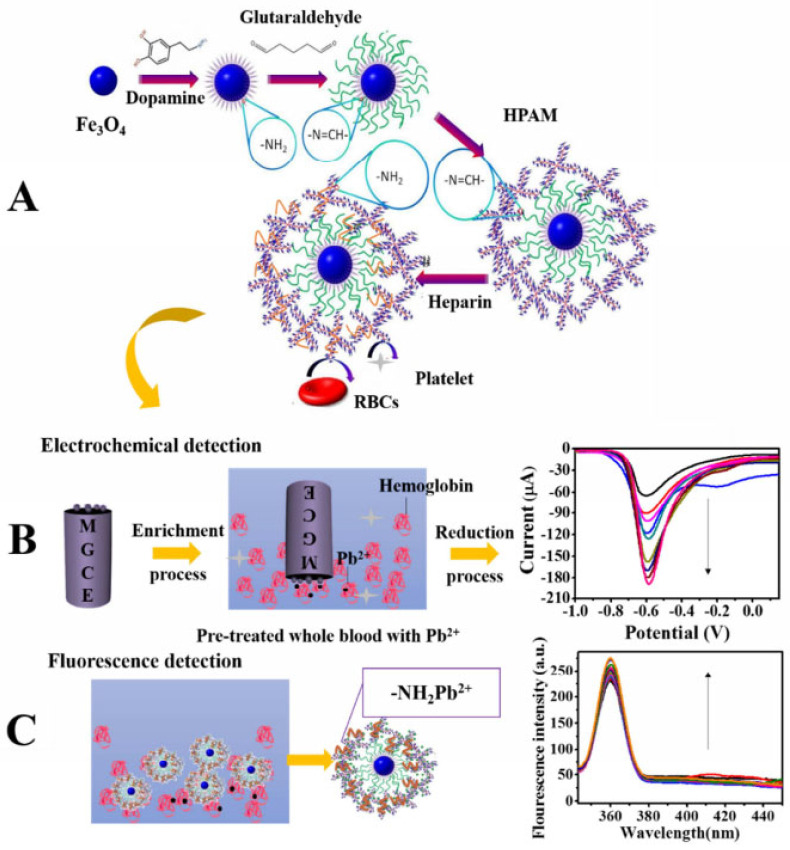
(**A**) Schematic illustration of the synthesis of MHPAM-H NPs and their applications in (**B**) the electrochemical detection and (**C**) the fluorescence detection for blood lead [54].

Chen et al. [54] reported a dual-function biosensor with electrochemical and fluorescent detection that can be used for blood lead detection. In this system, the presence of magnetic ferric oxide allows the sensor to be quickly fixed on the magnetic electrode. The surface of Fe_3_O_4_ is modified with hyperbranched polyamide (HPAM) with good fluorescence characteristics, rich amino groups, and cavity structure, which can form coordination bonds with lead ions to rapidly accumulate blood lead (Figure 5A). The enriched lead ions precipitate on the electrode surface and generate a current, while limiting the geometric movement of the fluorescence center of HPAM to enhance the fluorescence intensity. Based on this, the system realizes electrochemical and fluorescence dual-function detection of blood lead. The electrochemical detection range was 1.5–4.8 × 10^3^ pM and the detection limit was 4.4 pM Figure 5B. The fluorescence detection range was 0.5–4.8 × 10^3^ pM and the detection limit was 1.0 pM (Figure 5C).

**Figure 6:**
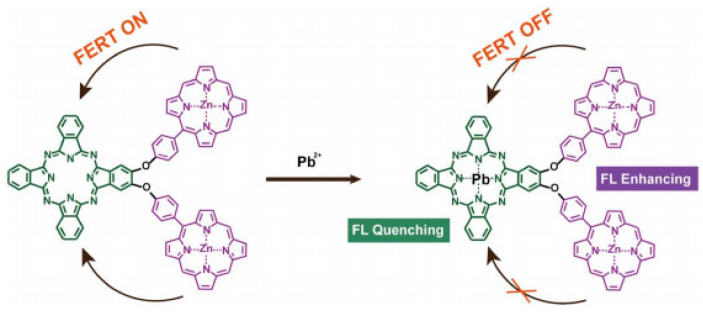
Lead ion detection mechanism [56].

Song et al. [55] synthesized a fluorescent fiber nanocrystal sensor for lead ion detection in aqueous solution, which is composed of fluorescent 1, 8-naphthimide dye covalently combined with cellulose nanocrystal (CNCs). The dye group grafted on the sensor and the adjacent carboxyl group on the surface of CNCs synergistically interact with lead ions in aqueous solution to show selectivity, resulting in a significant enhancement of fluorescence emission intensity. The binding ratio of lead ions to fluorophores on CNCs is 1.2:1 and the detection limit can be as low as 1.5 × 10^−7^ mol/L. Qi et al. [56] designed a fluorescence sensor of H_2_Pc-β-(ZnPor)_2_, a phthalocyanine porphyrin heterotriplet, for the determination of lead ions. This triplet has an efficient intramolecular fluorescence resonance energy transfer process (FRET) from two zinc porphyrin (ZnPor) units to a metal-free phthalocyanine (H_2_Pc) unit. Selective binding of lead ions to H_2_Pc effectively quench the fluorescence emission of the phthalocyanine unit (700 nm) while inhibiting the intramolecular FRET process and enhancing the fluorescence emission of the ZnPor unit (605 and 652 nm). A significant ratio fluorescence response was thus produced (Figure 6).

### 3.6. Electrode

Electrochemical analysis has the advantages of good sensitivity, large linear dynamic range, fast analysis time, and low cost. To realize the selectivity test of electrochemical method for lead ion in aqueous solution, a lead selective adsorption film is usually prepared to modify the electrode, then lead determination is carried out under certain conditions. Electrochemical analysis has the advantages of good sensitivity, large linear dynamic range, fast analysis time, and low cost. Under certain detection conditions, the electrochemical method usually realizes the selectivity test of lead ions in aqueous solution by preparing a lead-selective adsorption film to modify the electrode. As the electrode head is vulnerable to the interference of organic compounds, such as passivation and signal weakening, it is extremely important to pay attention to the reuse performance, precision, and anti-interference ability of the electrode while synthesizing high-sensitivity electrode modified films. Nguyen et al. [57] prepared a platinum nanoflower modified electrode by one-step electrochemical deposition method for simultaneous determination of lead ions and cadmium ions in water. Lead and cadmium ions in the range of 1–100 μg/L can be tested normally. The detection limits of the electrode for lead and cadmium were 0.408 and 0.453 μg/L, respectively. Guo et al. [58] established a method for determination of lead ions by square wave voltammetry using isoleucine modified glassy carbon electrode working electrode. Under the optimized conditions, the peak current Ip had a good linear relationship with lead ion concentration in the range of 5–50 nmol/L and 0.05–5.0 mmol/L, and the detection limit could be as low as 3.41 nmol/L. Although the newly fabricated electrode has high sensitivity, its reuse performance and anti-interference performance are not further described. In anodic stripping voltammetry, the lead detection signal is easily weakened or eliminated by surfactants and humic acid in environmental water. Grabarczyk et al. [59] found that adding a certain amount of Amberlite XAD-7 resin to the solution could eliminate the interference of some organic substances, such as surfactants without adsorbing lead ions, so that the electrode could directly detect lead ions in wastewater containing surfactants. A sensitive, rapid, and economical method for determination of lead in environmental water was developed. Silva et al. [60] used paraffin oil as the binder, cork, and graphite to synthesize a green, low-cost graphite/cork electrode material. Experiments show that the material has the best sensitivity to lead ions when the content of cork is 70% and the content of graphite is 30%. The sensitivity is better in 0.5 M sulfuric acid medium, and the detection limit of the method is 0.3 μM. Although the material is green and easy to synthesize, its sensitivity is relatively low compared with other methods, and acetate ion has a great influence on the determination of lead. Therefore, it can be further optimized. Deswati et al. [61] established an adsorbent cathodic stripping voltammetry (AdCSV) for the determination of lead and cadmium in seawater. In this method, calcium reagent was used as heavy metal complexing agent to adsorb lead ions and cadmium ions in seawater, then reduced on the surface of the hanging mercury drop electrode. Under the optimal testing conditions, the linear ranges of Pb and Cd were 10–160 ng/mL and 10–190 ng/mL, respectively, and the detection limits were 0.02 ng/mL and 0.05 ng/mL, respectively. The Pb and Cd showed good anti-interference performance against common ions in environmental water, but their re-use performance was unknown.

## 4. Comparison of Different New Detection Techniques

As the traditional dithizone color method consumes a large test dose, large waste liquid displacement, the masking agent used is highly toxic, and has a complicated operation, people continue to verify, improve, and supplement the methods of lead detection, so there is relatively mature atomic absorption spectrometry, atomic fluorescence spectrometry, electrochemistry, and other methods. Although the traditional method can achieve more accurate determination of lead in different fields, the greener, more efficient, and more portable method is still the main research focus. Among the new methods described in the review, The method based on precious metal nanotechnology has high sensitivity and can realize the detection of trace lead in environmental water samples. Paper-based microfluidic technology provides great convenience for on-site real-time detection, and its advantages of low cost, high sensitivity, non-professionals can use it, and quickly obtain test results make it have a huge development space and potential in the field of analysis and detection. The spectroscopic method is still the most commonly used method in the field of lead detection. It is the first choice in the field of lead detection because of its small amount of test samples, green and stable test links, and high detection accuracy. The electrochemical method also has good sensitivity, large detection linear range, low production cost, and high accuracy, which has become a research hotspot in the field of pre-detection. The summary of lead new measurement method is shown in Table 2.

The high sensitivity sensor based on noble metal nanoclusters relies on effective methods to synthesize excellent noble metal nanoclusters probes. The combination between ultra-small size (<2 nm) precious metals and lead ions is more sensitive. Therefore, the synthesis of stable and highly sensitive noble metal nanoclusters is a major difficulty in the field of precious metal nanotechnology in the detection of heavy metals. The design of paper chip structure is related to the detection efficiency, sensitivity, and specific selection performance. Therefore, how to optimize paper wetting conditions and liquid conveying speed to improve sample conveying capacity is the main research direction of this technology, and most of the studies in the review still have a long way to go from market application. Good photostability and high quantum yield have always been the focus of the research of fluorescent molecular probes. The reported synthesis of probe molecules is relatively complex. The design of a Pb^2+^ fluorescent probe with simple structure, low cost, good selectivity and high sensitivity has important research value and practical significance. The development of X-ray fluorescence spectroscopy, Fourier transform infrared spectroscopy, and so on are also developing towards the direction of low cost and high precision and are gradually applied in various fields of lead monitoring, but there are few applications in water quality monitoring. The change of environmental conditions, such as temperature and pH, will cause great interference to the spectral absorption and the electronic circuit of the instrument, which makes it difficult to meet the accurate measurement of different water quality. Electrochemical method of sensitization of nanomaterials, more green synthetic raw materials, and other detection of lead ions in water has shown a wider range of application, but electrode film activity and preservation stability are still a major difficulty of electrode method, especially the preservation of nanomaterials is extremely unstable, they will become inefficient due to degeneration, degradation, or aggregation. This will greatly reduce the detection performance of the sensor.

## 5. Conclusions

Lead ions can easily enter the human body through contaminated water and food chain. Due to the non-degradability of lead, it is easy to accumulate in animals and plants. In severe cases, lead can lead to systemic diseases in humans, and ultimately pose a great threat to human health. The high content of lead in the environment can reduce the photosynthetic rate of plants and crop production. Therefore, it is imperative to accurately monitor the lead pollution in people’s daily living environment. This paper briefly introduces the harmful effects of lead ions and the emission limits of lead ions in various fields. The national standard detection methods of lead ion, such as atomic absorption spectrophotometry, electrochemical method, inductively coupled plasma mass spectrometry, etc., are introduced. There are many methods for lead ion detection, and their applicable fields and test ranges are also different. Each method has its own advantages and disadvantages. Thus, in the actual test process, we should choose a more suitable test method according to different test conditions. When the content of lead in the sample and the sample volume are small, the spectral and electrochemical methods can be used to determine the content of lead. When the sample quantity is large or there is not enough detection budget, the dithizone color method can be selected for determination. A safer, greener, and more efficient pre-treatment technology is also one of the hot topics in the field of lead ion detection due to the complicated pre-treatment process during sample testing.

With the progress of science and technology, emerging detection technologies, such as the application of precious metal nanotechnology, paper-based microfluidic technology, synthesis technology of new fluorescent molecular probes, and new green and efficient electrochemical technology, also emerge in an endless stream.

Due to the high price of traditional detection technology and the complex extraction technology with a certain toxicity, the research of various new technologies is constantly developing in the direction of more economic, more portable, and more rapid. The future research directions of lead detection can be as follows: first, the direction of higher detection efficiency and measurement accuracy. The second is to reduce the pollutants produced in the experiment and use as few samples as possible to get as high accuracy as possible. The third is to develop towards a wider range of applications, such as air, soil, biomedicine, and so on. Fourth, it can be developed in the direction of reducing equipment costs, realizing real-time monitoring, and more portable and efficient testing. Therefore, the development trend of lead ion detection technology in the future must be lower equipment cost, more portable operation, more rapid and efficient, better stability, and higher accuracy.

## Figures and Tables

**Table 1:** Comparison of traditional detection methods for lead ions.

Methods		Limit of Detection	Advantages	Disadvantages
spectrometry	Atomic absorption spectrophotometry/ atomic fluorescence spectrometry/ inductively coupled plasma atomic emission spectrometry	≤2.5 μg/L [19] 5 μg/L [24] 0.2 mg/L (direct determination) and 2 μg/L (after chelation and extraction) [25]	Low detection limit, high accuracy, good selectivity, less sample consumption, and a wide range of applications.	The testing equipment is expensive, and it is highly interfered by matrix
colorimetric method	Dithizone method	0.01 mg/L [19]	General laboratory can realize the determination of lead, and the detection cost is low	The detection procedure is more complicated, KCN masking agent is highly toxic, and the presence of a large amount of tin interferes with the determination. The amount of waste liquid produced is large
electrochemical method	Oscorography polarography/ voltammetry	0.01 mg/L [19] 0.1 μg/L [23]	The operation is simple and the equipment price is relatively cheap, less waste liquid is produced.	Water samples containing Sn^2+^ and As^3+^ require additional acid or pretreatment

**Table 2:** Summary of lead new measurement method.

Methods		Concentration Range	Applicable Objects	Advantages	Disadvantages
Precious metal nanotechnology	AgNPs/PVA [39] MSA-capped GNP-supported alumina [40]	20–10,000 μg/L [39] 0–50 μg/L [40]	Surface water and industrial waste water [39,40]	High sensitivity	It is difficult to synthesize and has a poor shelf life
Paper-based microfluidic technology	Chromate method [46] Sodium rhodizonate method [47] AuNPs/Glu method [48]	1.37–175 μg/L [46] 10–100 μg/L [47] 0–1000 μg/L [48]	Environmental water and industrial waste water [46,47,48]	It has low cost, real-time monitoring on site, and can be used by non-specialists	The sensitivity was low, and the paper chip structure design did not meet the market demand
Spectrometry	CO_2_ laser induced spectroscopy [51] Chemometrics-Based Fourier Transform Infrared Spectroscopy [52]	>6 wt.-% [51]	Glass [51] Blood lead [52]	High precision, wide application field	Due to the large interference, it is difficult to meet the accurate measurement of different water quality. High cost
Fluorescent molecular probe	A dual-responsive biosensor [54] Fluorescent cellulose nanocrystals [55]	0.5–4.8 × 10^3^ pM [54] 2.5 × 10^−7^–5.0 × 10^−5^ mol/L [55]	Blood lead [54] chemical, environmental, and biological systems [55].	Good sensitivity, high selectivity, and short response time	The synthesis is complex and the photostability is unknown
Electrode	Anodic Stripping Voltammetry [59] Graphite/Cork sensor [60] Adsorptive cathodic stripping voltammetry [61]	2 × 10^−9^–5.0 × 10^−6^ mol/L [59] 1–25 μmol/L [60] 10–160 ng/mL [61]	Waste water [59] Natural water [60] Seawater [61]	Good sensitivity, large linear dynamic range, fast analysis time, and low cost	The electrode membrane is easily inactivated by interfering substances, and the shelf life needs to be improved

## Data Availability

The data that support the findings of this study are available from the corresponding author upon reasonable request.
